# Differences at brain SPECT between depressed females with and without adult ADHD and healthy controls: etiological considerations

**DOI:** 10.1186/1744-9081-5-37

**Published:** 2009-09-01

**Authors:** Ann Gardner, Dario Salmaso, Andrea Varrone, Alejandro Sanchez-Crespo, Susanne Bejerot, Hans Jacobsson, Stig A Larsson, Marco Pagani

**Affiliations:** 1 Karolinska Institutet, Department of Clinical Neuroscience, Section of Psychiatry, Karolinska University Hospital Huddinge, Stockholm, Sweden; 2 Institute of Cognitive Sciences and Technologies, CNR, Rome & Padua, Italy; 3 Karolinska Institutet, Stockholm Brain Institute, Department of Clinical Neuroscience, Psychiatry Section, Karolinska Hospital, Stockholm, Sweden; 4 Department of Nuclear Medicine, Karolinska University Hospital, Stockholm, Sweden; 5 5 Karolinska Institutet, Department of Clinical Neuroscience, Section of Psychiatry, St Goran´s Hospital, Stockholm, Sweden; 6 Department of Radiology, Karolinska University Hospital, Stockholm, Sweden

## Abstract

**Background:**

Comorbidity between Attention Deficit Hyperactivity Disorder (ADHD) and mood disorders is common. Alterations of the cerebellum and frontal regions have been reported in neuro-imaging studies of ADHD and major depression.

**Methods:**

Thirty chronically depressed adult females of whom 16 had scores below, and 14 scores above, cut-offs on the 25-items Wender Utah Retrospective Scale (WURS-25) and the Wender-Reimherr Adult Attention Deficit Disorder Scale (WRAADDS) were divided into subgroups designated "Depression" and "Depression + ADHD", respectively. Twenty-one of the patients had some audiological symptom, tinnitus and/or hearing impairment. The patients were investigated with other rating scales and ^99m^Tc-HMPAO SPECT. Controls for ^99m^Tc-HMPAO SPECT were 16 healthy females. SPECT was analyzed by both statistical parametric mapping (SPM2) and the computerized brain atlas (CBA). Discriminant analysis was performed on the volumes of interest generated by the CBA, and on the scores from rating scales with the highest group differences.

**Results:**

The mean score of a depression rating scale (MADRS-S) was significantly lower in the "Depression" subgroup compared to in the "Depression + ADHD" subgroup. There was significantly decreased tracer uptake within the bilateral cerebellum at both SPM and CBA in the "Depression + ADHD" subgroup compared to in the controls. No decrease of cerebellar tracer uptake was observed in "Depression". Significantly increased tracer uptake was found at SPM within some bilateral frontal regions (Brodmann areas 8, 9, 10, 32) in the "Depression + ADHD" subgroup compared to in "Depression". An accuracy of 100% was obtained for the discrimination between the patient groups when thalamic uptake was used in the analysis along with scores from Socialization and Impulsivity scales.

**Conclusion:**

The findings confirm the previous observation of a cerebellar involvement in ADHD. Higher bilateral frontal ^99m^Tc-HMPAO uptake in "Depression + ADHD" compared to in "Depression" indicate a difference between these subgroups. ^99m^Tc-HMPAO uptake mechanisms are discussed.

## Background

The persistence of Attention Deficit Hyperactivity Disorder (ADHD), previously thought of as a self-limiting condition that rarely requires treatment in adult life, has only come into focus of widespread clinical attention and research in the last decade. In 1998, it was suggested that ADHD may be "the most common chronic undiagnosed psychiatric disorder in adults" [1]. According to a U.S. survey estimation, the prevalence of adult-ADHD may be as high as 4.4% [2]. Higher medical costs and more absences from work are associated with ADHD in adults [3]. Lifetime mood disorder was reported by 53% of the patients with adult-ADHD, versus in 28% of the controls, in a study [4]. It has been suggested that the complex emotional symptoms in females with adult-ADHD may obscure the diagnosis of ADHD [5]. An earlier age of onset of major depression, and a comorbidity of 87% with at least one other psychiatric disorder, was reported in a study of adult-ADHD subjects compared with non-ADHD subjects [6]. For the psychiatric and somatic comorbidity with adult-ADHD, see [Additional file 1].

High heritability of ADHD irrespective of whether continuum or categorical approaches were used, or if different cut-off criteria were applied, was reported in a large twin study [7]. The requirement of specific numbers of symptoms to be present may impose an artificial categorical construct. Sachdev has suggested that adult-ADHD should be studied without restricting the examination to those meeting some arbitrary diagnostic criteria [8].

Similarities between adult and childhood ADHD such as impaired cerebello-(thalamo)-striato-frontal networks have been suggested [9]. In a study of magnetic resonance imaging (MRI) in adult-ADHD, there was significantly smaller volumes of the overall cortical grey matter, and prefrontal and anterior cingulate cortex, while total cerebral volume was normal, compared to healthy controls [10]. Decreased global cerebral glucose metabolism in adult-ADHD at Positron Emission Tomography (PET), with regional reductions in several regions including the premotor and the superior prefrontal cortex, has been reported [11]. Less activation at decision making in adult-ADHD at PET using O-15 labeled water was observed in brain regions including Brodmann area (BA) 32 in the anterior cingulate cortex [12].

Single Photon Emission Computed Tomography (SPECT) is the mostly available functional neuro-imaging method. SPECT systems detect γ-rays emitted by injected radioactive substances.

The uptake of the radiotracer ^99m^Tc-*d,l*-hexamethylpropylene amine oxime (^99m^Tc-HMPAO) used at SPECT is generally considered to reflect cerebral blood flow (CBF). However, discrepancies in the same brains have been reported between the uptake of ^99m^Tc-HMPAO and other SPECT perfusion tracers, and with PET results [13-15]. Such differences may be related to the specific retention mechanism of ^99m^Tc-HMPAO [13].

### The aims of the present study

The main aim of the study was to identify any regional CBF differences as reflected by the brain uptake of ^99m^Tc-HMPAO in chronically depressed females subdivided for the absence or presence of adult-ADHD, and in healthy controls. The second aim was to find etiological models for any emerging differences. The third aim was to identify factors accounting for most of the differences between the patient groups.

## Materials and methods

### Subjects

Subjects were 30 adult females (ages 26-58 years, mean 43 ± 10) attending a specialized tertiary psychiatric hospital-affiliated out-patient unit for clients with any type of audiological symptom and their relatives in need of psychiatric care. All subjects had an ongoing chronic (at least two years) depressive disorder fulfilling the DSM-IV criteria for major depression [16] at least once. In 21 patients there was tinnitus, hyperacusia, vertigo, and/or hearing impairment. The hearing impairment was severe with childhood onset in five patients. Nine patients without any audiological symptoms had first-degree relatives with audiological and psychiatric symptoms. Exclusion criteria were austistic disorders, bipolar disorder, psychosis, antisocial personality disorder, alcohol or other substance abuse/dependence, or serious medical illness. Eighteen patients (60%) were free of any psychiatric medication at the time of the SPECT scan apart from sleeping agents in some patients. Nine patients (30%) were medicating with antidepressant agents, and three patients with anxiolytic agents (10%). Two of the antidepressant-medicated patients had other ongoing medication, in one case quetiapin, in the other modafinil. The SPECT scans were obtained at an out-patient basis in all patients when they were in their habitual state and not during episodes of increased mood symptomatology.

### ADHD-questionnaires

The patients were subgrouped according to their self-rated scores on the 25-items version of the Wender Utah Retrospective Scale (WURS-25), and the Wender-Reimherr Adult Attention Deficit Disorder Scale (WRAADDS). A cut-off score of 36 for WURS-25, which has a 5-point response format, was applied since it had been shown to correctly identify 96% of an ADHD group, and 96% of the non-patient comparison group in a study [17]. Current adult-ADHD-like symptoms were assessed with the 35-items 5-point response format WRAADDS [5]. The WRAADDS has seven subscales: Attentional Difficulties, Hyperactivity/Restlessness, Temper, Affective Lability, Disorganization, Stress Sensitivity, and Impulsivity. The instruction for the WRAADDS questionnaire was changed from "during all your adult life" into "during the past years". A cut-off score of 50 was chosen since depressed patients with lower scores usually deny any clinically significant cognitive symptoms according to clinical experience. The results of the questionnaires were presented to the patients who then were interviewed about past and current ADHD symptomatology. Scores below cut-offs are referred to as "low", and at/above cut-offs as "high" in the following text.

### Mood and personality questionnaires

The self-rated 9-items version of the Montgomery Aasberg Depression Rating Scale with a 6-point response format [MADRS-S; [18]], was used to assess current mood at SPECT. The 20-items Socialization subscale from the self-rated Karolinska Scales of Personality (KSP) with a 4-point response format was used to assess the dimensions of alienation, victimization, resentment and dysphoria. The KSP Socialization scale has been adapted from the Swedish translation and psychometric analysis of the California Psychological Inventory (CPI) Socialization scale and contains items dealing with negative childhood experiences [19]. The Socialization scale possesses remarkable stability over time both in a population sample [20] and in depressed patients [21]. The scores of the patients were compared with normative data transformed to T-scores (50 ± 10) obtained from 200 females randomly sampled from the Stockholm population and standardized for gender and age [19]. Low scores at Socialization indicate pathology.

### Neuropsychological investigation

Five patients with high WURS-25 and WRAADDS scores, all adult daughters with normal hearing of hearing impaired mothers, were thoroughly investigated with a neuropsychological test battery at a specialized neuropsychiatric unit. The investigation included clinical interviews (in four cases also with mother/parents), questionnaires, rating scales, and neuropsychological tests including the computerized visual Test of Variables of Attention [T.O.V.A.; [22]].

### Healthy controls for SPECT

Sixteen healthy adult females (ages 25-56 years, mean 40 ± 13), all with normal MADRS scores, served as controls. They were selected from a group of individuals who had undergone SPECT with the intention that they would be utilised as a normal control group for SPECT analyses [23]. The study was approved by the local Ethics and Radiation Safety Committees (Karolinska sjukhuset Nord 97-141; KI(Syd)HS 423:00; Regionala etikprövningsnämnden 04-1079/1; Strålskyddskommittén, Karolinska Universitetssjukhuset, Solna, Stockholm 03/05). All subjects gave written informed consent.

### SPECT

Brain imaging using SPECT was performed using a three-headed Gamma Camera (TRIAD XLT 20, Trionix Research Laboratory Inc., Twinsburg, OH, USA) equipped with low-energy ultrahigh-resolution (LEUHR) collimators. The intrinsic spatial resolution of the camera was 8 mm at Full Width Half Maximum. ^99m^Tc-HMPAO (Ceretec^®^, Exametazine, Amersham International Plc, Little Chalfont, UK) was injected after 30 minutes rest in a quiet dim lighted room. Examinations started between 45 and 60 minutes after tracer injection. The projection data were acquired for 30 seconds per projection at 90 equal angles of a complete revolution. Between 8 and 10 million total counts were acquired.

Before back-projection we pre-processed the one-dimensional data with a Hamming smoothing filter with a cut-off frequency of 2.25 cycles/cm. Then SPECT images were reconstructed by filtered back projection algorithm using a ramp filter with a cut-off frequency of 0.6 cycles/cm. Attenuation correction was based on a four-point ellipse [24]. No scatter correction was performed. Data were projected into a 128 × 128 pixel matrix resulting in an isotropic voxel size of 2.2 mm^3^.

### SPM2 analysis

SPECT raw images were transformed into the analyze format by XMedCon package. Data were analyzed with SPM2 (Wellcome Department of Cognitive Neurology, London, UK) implemented in Matlab 6.5.1. Images of relative tracer distribution were spatially normalized into the stereotactic Montreal Neurological Institute (MNI) space, a predefined SPECT template available in SPM2 (voxel size 2 × 2 × 2 mm), using a 16-parameter affine (non-linear) transformation. Because this template doesn't completely match the Talairach brain, it was necessary to correct the SPM{t} coordinates. This was achieved using the subroutine implemented by Matthew Brett [25]. BAs where then identified within a range of 3 mm, after importing the corrected coordinates, by the Talairach Daemon Database [26].

After normalization, images were smoothed with a Gaussian filter of 12 mm (FWHM) to account for individual gyral differences and brain anatomy and to increase the signal-to-noise ratio. Images were globally normalized for signal intensity using proportional scaling to remove confounding effects due to global CBF changes, with a threshold masking for grey matter of 0.8, allowing to include into the analysis only those voxels whose intensity exceeded the 80% of the maximal one.

The voxel-based analyses were performed using SPM2 with a "one scan per subject, Ancova" design model, and significances were sought for the contrasts between healthy controls and the patient groups, and between the patient groups.

### CBA analysis

For non-voxel based evaluation and analysis, the SPECT data were analyzed by a computerized brain atlas (CBA; Applied Medical Imaging AB, Uppsala, Sweden) [27] allowing for identification of various anatomical brain structures. All image sets were spatially normalized into the stereotactic space of the atlas by using the global polynomial transformation. It consists of translations, rotations and linear scaling along and around each of the three image axes. It also contains 18 nonlinear shape-deforming parameters, which make it possible to individualize the brain shape. Then when needed, further manual adjustments were performed to optimize the fitting of the CBA contours to both external and internal (brain ventricles) borders. In order to obtain a set of normalized relative metabolic data, a scaling factor was computed averaging all brain voxels data and setting the global brain average to the conventional value of 50 'uptake units'. All regional uptake values were then related to this value. Normalized tracer uptake for 54 volumes of interest (VOIs), 27 from each hemisphere, was automatically assessed by the CBA. Each VOI was automatically built up by summing the corresponding regions of interest in consecutive sections. The selected VOIs were thalamus, nucleus caudatus, putamen, insular region, hippocampus, cerebellum, and BAs from the frontal, temporal, parietal, and occipital lobes, and the cingulate cortex.

### Discriminant analysis

Stepwise discriminant analysis was performed to compare clinical diagnosis as determined by the ADHD questionnaires and interview to the grouping according to some of the variables under investigation. This exploratory analysis was especially performed in order to identify the diagnostic factors accounting for the most of the group differences. The outcome of discriminant analysis resulted in discriminant functions (DF) that are the linear combinations of variables included in the analysis. The relevance of a DF is given by its canonical correlation (R), i.e., the total variance explained by each DF (R, 0-1). On the basis of the scores computed for each subject, a classification matrix was computed to evaluate the efficiency of the analysis. Cases were classified as belonging to the group where the highest classification score was reached. Significance of the discriminant analysis was tested with aprox-F value.

### Statistical analysis

A threshold of p < 0.01 was set for the group differences on demographic data and rating scores. For group differences at SPM2, thresholds of p = 0.01 uncorrected for multiple comparisons for voxel height, p_corrected _< 0.05 at cluster level and p_uncorrected _< 0.001 at voxel level were chosen. At voxel level, the false discovery rate (FDR) method for multiple comparison correction was also explored. In this latter case voxels were considered significant at a p_corrected _< 0.05. Only those clusters containing more than 200 voxels were accepted as significant. This was based on the calculation of the partial volume effect resulting from the spatial resolution of the camera system. The significance threshold for the CBA analyses was set at p < 0.05.

## Results

### Subgrouping of patients according to ADHD-questionnaires and interview

The patients with low WURS-25 and WRAADDS scores were designated as the "Depression" subgroup (N = 16), and the patients with high WURS-25 and WRAADDS scores as the "Depression + ADHD" subgroup (N = 14). The patients endorsed the absence or presence of the ADHD symptoms as assessed by the questionnaires at the interview subsequent to filling in the ADHD-questionnaires. Two of the five patients with early hearing impairment were included in the "Depression" subgroup, and three in the "Depression + ADHD" subgroup. Three of the nine patients without audiological symptoms were included in "Depression", and six in "Depression + ADHD".

### Neuropsychological investigation

The results of the thorough investigation of five patients included in the "Depression + ADHD" subgroup confirmed ADHD in three patients, and the predominantly inattentive subtype, Attention Deficit Disorder (ADD), in two patients.

### Demographic and other data in the patient groups

Demographic and other data are presented in Tables 1 + 2. Since age differed between the patient subgroups, it was considered as a nuisance variable in the comparisons between "Depression + ADHD" and "Depression" at both SPM2 and CBA. There were no proportional significant differences for work capacity, co-habitation with a partner, motherhood, or medication at SPECT. The "Depression + ADHD" patients were significantly younger at their own estimation for first onset of mood symptoms. There was no significant difference for years spent since onset of mood symptoms ("Depression": 14 ± 12 years, "Depression + ADHD": 19 ± 12 years, p = 0.237). The scores at the depression rating and Socialization were significantly more abnormal in the "Depression + ADHD" subgroup.

**Table 1:** Demographic and other data

Subgroup/p-values	No of cases	Age	WURS-25	WRAADDS < 40 considered normal ≥ 70 indicates ADHD	Socialization 50 ± 10 normal (low score abnormal)	Age at first onset of mood symptoms (patients' own estimation)
"Depression"	16	47 ± 9	15 ± 8	37 ± 11	43 ± 10	33 ± 12
"Depression + ADHD"	14	39 ± 9	59 ± 18*	89 ± 16	22 ± 10**	18 ± 13
*t*-test p-value		0.029	0.0000	0.0000	0.0000	0.004

*The WURS-25 score in one patient and included here: 31 (as assessed by mother: 42). **13 patients.

**Table 2:** Demographic and other data

Subgroup/p-values	No work capacity	Not married or living with partner	No children	MADRS-S	Anti-depressant medication	Anxiolytic medication
"Depression"	12/16	7/16	3/16	16 ± 6	3/16	1/16
"Depression + ADHD"	10/14	9/14	6/14	28 ± 11	6/14	2/14
*t*-test p-value				0.0008		
χ^2^-test p-value	0.825	0.261	0.151		0.151	0.464

### Comparisons of the tracer uptake at SPECT between controls and patients

**Figure 1:**
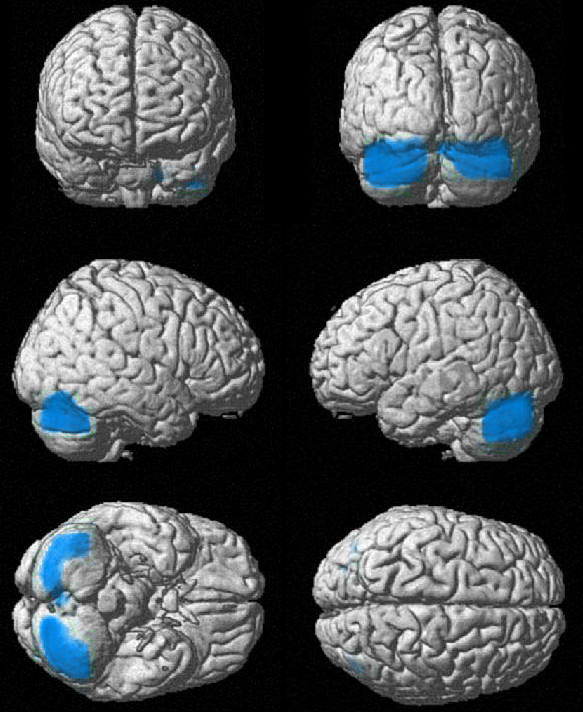
**Voxels reflecting lower tracer uptake (blue) in the adult-ADHD depressed patients compared to the healthy controls**. Left row from top to bottom: uptake in the left cerebellum in as seen at a frontal view of the brain; in the right cerebellum at the side view of the right hemisphere; and in the bilateral cerebellum at the bottom view. Right row: uptake in the bilateral cerebellum seen at the back view of the brain; in the left cerebellum at the side view of the left hemisphere; and as barely seen (and non-significant) in the bilateral parieto-occipital lobe at the top view of the brain.

The SPM subtraction for the healthy controls minus "Depression + ADHD" revealed significantly decreased tracer uptake within the bilateral cerebellum in the patient group (p_corrected _= 0.024 at cluster-level and p_uncorrected _< 0.001 at voxel-level), see Figure 1. No significant differences were found at the opposite comparison. When the controls were compared to "Depression", no significant changes were found at both subtractions.

At the CBA evaluation, an Hemisphere*VOIs*GROUP interaction (F(26,728) = 1.749, p < 0.05) was found. There was decreased tracer uptake in the "Depression + ADHD" subgroup compared to controls in the bilateral cerebellum (F(1,28) = 13.429, p < 0.001) and in the caudate nuclei (F(1,28) = 10.013, p < 0.01).

### Comparisons of the tracer uptake at SPECT between the patient groups

**Figure 2:**
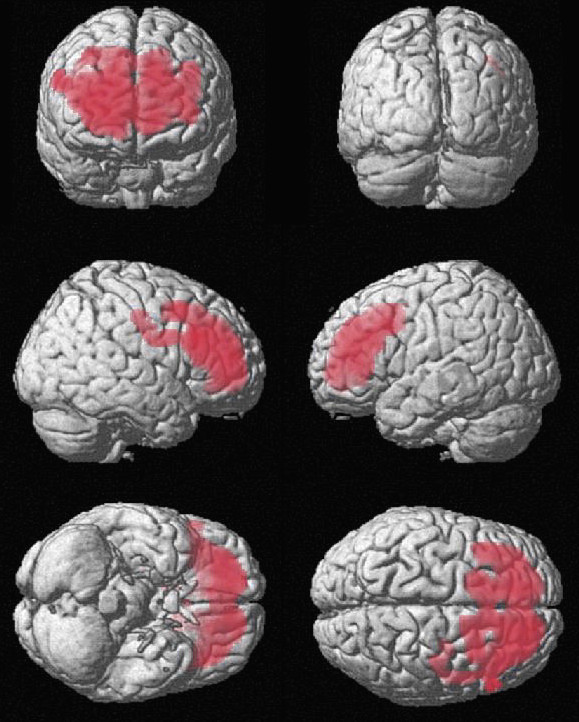
**Voxels reflecting higher tracer uptake (red) in the adult-ADHD depressed patients compared to the depressed patients**. Left row from top to bottom: uptake in the frontal lobe as seen at a frontal view of the brain; in the right frontal lobe at the side view of the right hemisphere; and in the orbito-frontal lobe at the bottom view. Right row: the back view of the brain; in the left frontal lobe at the side view of the left hemisphere; and in the bilateral frontal lobe at the top view of the brain.

**Figure 3:**
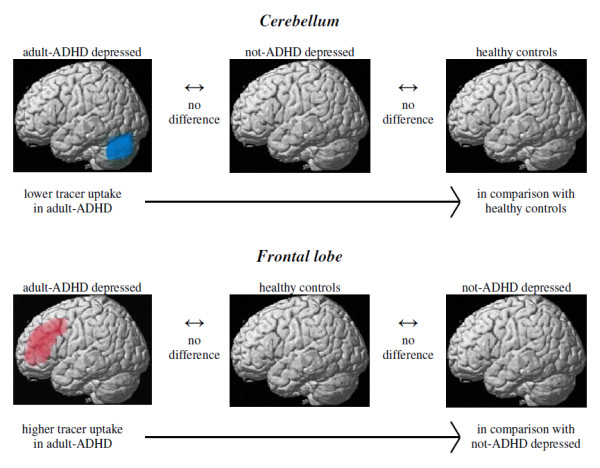
**The differences of the ^99m^Tc-HMPAO uptake at SPECT as analyzed by SPM2 between the adult-ADHD depressed patients and the other groups are exemplified by left hemisphere images**.

The SPM subtraction for the "Depression + ADHD" minus "Depression" revealed a large cluster of highly significantly increased tracer uptake (p_corrected _< 0.001 at cluster-level; p_uncorrected _< 0.001 and p_FDR-corrected _= 0.013 at voxel level) in "Depression + ADHD" within the bilateral BA 32 in the anterior cingulate cortex, in BAs 8, 9 and 10 in the prefrontal and middle frontal cortex, and in large portions of the frontal lobe white matter, see Figure 2. No significant differences were found at the opposite comparison. The SPM findings are summarized in Figure 3.

When implementing the CBA in the "Depression + ADHD" versus "Depression" comparison, there was a significant VOIs*GROUP (F(26,728) = 1.605, p < 0.05) interaction. Tracer uptake was significantly decreased in "Depression + ADHD" as compared to "Depression" in the putamen (F(1,28) = 6.525, p < 0.05) and in the thalamus (F(1,28) = 5.209, p < 0.05).

### Discriminant analysis

In discriminating between "Depression" and "Depression + ADHD", the WRAADDS subscale Impulsivity, the KSP subscale Socialization, and the thalamic tracer uptake were found to be the most useful variables. The 6-items Impulsivity subscale constitutes 17% of the WRAADDS items (mean scores "Depression": 3 ± 2, "Depression + ADHD": 15 ± 4, p < 0.0001). Results for Socialization are presented in Table 1. Using these variables, the percentage of correct classification as compared to clinical diagnosis reached 100% in both sensitivity and specificity (Aprox.F(3,25) = 49.42, p < 0.001). The canonical correlation was 0.925 yielding a 85.6% of total variance explained by our grouping.

## Discussion

In this study we report decreased ^99m^Tc-HMPAO uptake in the cerebellum at SPECT in chronically depressed females with adult-ADHD. No decrease of cerebellar ^99m^Tc-HMPAO uptake was observed in chronically depressed females without ADHD. A relative increase of tracer uptake mainly in the frontal and anterior limbic cortex was found in the "Depression + ADHD" subgroup as compared to in "Depression". Self-rated scores for ADHD symptomatology followed by clinical interview were used to divide all the patients into subgroups with likely absence or presence of adult-ADHD. The DSM-IV-criteria for ADHD [16] were not applied since they refer to ADHD without concomitant mood disorder. The diagnosis of adult-ADHD/ADD was supported in those patients in whom neuropsychological evaluations were performed by a specialized team.

SPECT provides image processing, tomographic reconstruction and image display. SPECT has a lower spatial resolution as compared to PET (8-10 mm versus 4-7 mm) and does not allow for quantitative assessment. Nevertheless, brain SPECT reflects CBF and neuronal activity distribution, resulting in reliable brain mapping with the use of radiopharmaceuticals that are more easily obtainable commercially than those applied at PET, making SPECT the most common functional imaging technique in routine examinations. Time resolution for perfusional SPECT is between two and three minutes, being lower than fMRI or methodologies based on magnetic activity of the brain (MEG). Image acquisition can be started up to some hours after administration still representing the brain distribution of the radiopharmaceutical at the moment of injection allowing for experiments to be performed in ideal psychological conditions in a quiet environment outside of the camera gantry.

SPECT was analyzed with SPM and CBA, three-dimensional digitized spatial standardization software which approach statistical analysis from different points of view. SPM implements univariate analysis creating *t*-statistic based maps. CBA utilises the data from regions or VOIs able to feed multivariate analysis enabling the investigation of more general statistical effects. SPM analysis is conducted at cluster of voxel level while the CBA produces data at the larger VOI level. SPM does not usually analyse regions defined *a priori *and identifies changes only if clustering in a number of voxels exceed the determined threshold, often missing general effects. Conversely CBA cannot analyse clusters of voxel smaller than the pre-defined VOIs generally failing to detect fine regional changes. It is thus not surprising that the two methods might highlight different topographic changes in different regions. Hence, the double analysis underscores their complementarity in identifying global and local functional changes.

Decreased uptake (and retention) of the tracer ^99m^Tc-HMPAO reflects various phenomena such as decreased blood flow, loss of cells or cellular constituents, decreased neuronal activity in functional circuits, and/or the metabolic status of the tissue. There is no overt linear correlation between the uptake of ^99m^Tc-HMPAO and CBF as measured at PET using O-15 labeled water in healthy subjects [28,29]. Uncoupling between the ^99m^Tc-HMPAO uptake and the cerebral glucose metabolic rate at PET has been reported in the left basal ganglia in depressive disorder [15]. Measured change in local brain activity reflects both physiological processes and neuroenergetic mechanisms. The mechanism by which ^99m^Tc-HMPAO is intracellularly trapped and retained is also related to the metabolic state. ^99m^Tc-HMPAO uptake can parallel the cellular content of reduced glutathione (GSH) [30], but the overall retention of ^99m^Tc-HMPAO may be more dependent upon the redox activity than GSH content only [31]. GSH is involved in the cellular redox balance and in the protection against oxidative damage. In disorders of the cellular energy production in mitochondria, an increased cellular GSH content may occur initially in the disease [32]. The decreased cellular GSH content that was reported in a subset of patients with such conditions may occur as a consequence of the increasing oxidative stress that can appear with time [33].

At the comparisons between the "Depression + ADHD" subgroup and controls, decreased tracer uptake within the cerebellum was found at both SPM and CBA in the patients. The cerebellum is involved in cognitive and behavioural control and emotional processing. Lesions in parts of the cerebellum are hypothesized to modulate actions via a "dysmetria of thought" [9]. Decreased cerebellar activity has been observed at functional MRI (fMRI) in adult-ADHD [34].

In a longitudinal MRI study of children with ADHD, there were smaller volumes of the cerebellar vermis, and changes over time of the total cerebellar volume in the subgroup with worse outcome [35]. A cerebellar over cerebral involvement as detected by MRI has been reported in a quarter of patients with heterogeneous disorders affecting the neuroenergetic metabolism in mitochondria [36]. The cerebellar hemispheres and vermis are affected particularly in the mitochondrial disorder associated with deficiency of Q10, a component of the respiratory chain which acts as an antioxidant because of its ability to transfer electrons [36]. Chronic administration of methylphenidate (MPD), considered to be the first-line drug in adult-ADHD, increases the activities of mitochondrial respiratory chain complexes in the cerebellar vermis [37]. MPD binds to dopamine transporters implicated in the vulnerability to ADHD which are expressed also in the cerebellum, and blocks the inward transport of dopamine [37,38]. MPD responders among ADHD patients have a high availability for dopamine transporters in contrast to nonresponders [39]. Since dopamine has been shown to inhibit complex I of the mitochondrial respiratory chain [40], the pathophysiology of ADHD may involve mitochondrial dysfunction which by itself has been shown to exert effects on dopamine turnover [41,42], and/or relate to primary alterations of dopamine transporters.

In a postmortem specimen study of major depression, decreased expression levels of subunits of mitochondrial complex I were found in the cerebellum. There were no alteration in any other of the analyzed brain region. A less consistent pattern of cerebellar reductions was observed in bipolar disorder along with increased subunit expression in the parieto-occipital cortex [43]. In light of the high comorbidity between mood disorders and adult-ADHD [4-6], the cerebellar alterations in this [43] as well as in a study of recovered patients with recurrent major depression [44] might reflect adult-ADHD in a proportion of the subjects. The decreased tracer uptake in the cerebellum in the "Depression + ADHD" subgroup may hypothetically reflect some longstanding process preferentially affecting this brain region involving oxidative stress and mitochondrial energy production, and/or relate to the higher depression severity in "Depression + ADHD".

Increased tracer uptake at SPM in "Depression + ADHD" was found at the comparisons with "Depression" in BA 32 in the bilateral anterior cingulate cortex, BAs 8, 9 and 10 in the prefrontal and middle frontal cortex, and adjacent white matter. An increase of white matter volume has been reported in adult-ADHD [10]. The anterior cingulate is considered to play a critical role in complex cognitive processing, particularly target detection, response selection, error detection, and reward-based decision-making, functions that are thought to be impaired in ADHD [10]. Signs indicating functional *hypoactivity *in adult-ADHD at PET [12] and fMRI [45] have been reported for the anterior cingulate cortex, and for the premotor cortex and the superior prefrontal cortex [11]. Failure to suppress activity in the default-mode network in the ventral medial prefrontal cortex and posterior cingulate cortex, and precuneus in the midline, has been suggested as the neural substrate of ADHD-relevant behaviour [46]. Time series fMRI has demonstrated strong evidence of disconnection between an anterior control region and posterior components of this network [46].

In a metaanalysis of neuro-imaging studies of major depression, "maximally abnormal" loci were identified within BA 32 and 24 in the anterior cingulate, and at the border of BAs 46 and 9 in the prefrontal cortex. Although more studies were categorized as reporting "overactivity", the fraction was not significant [47]. However, the increased tracer uptake in the "Depression + ADHD" subgroup as compared to in "Depression" in BA 32 in the anterior cingulate cortex and BAs 8, 9 and 10 in the prefrontal and middle frontal cortex may not be related to the mood differences between these groups since no difference of the tracer uptake was found between controls and "Depression", or between controls and "Depression + ADHD", in these regions.

Decreased tracer uptake in the frontal lobe has been reported in most ^99m^Tc-HMPAO SPECT studies of depression. Increased frontal lobe tracer uptake has been reported in studies of atypical and chronic depression [48]. Increased frontal lobe tracer uptake has also been described in depressed patients with decreased muscle activities of mitochondrial enzymes in comparison with depressed patients with normal enzyme activities [49]. Compensatory strategies eliciting increased cognitive (prefrontal, BAs 8 and 9) control were suggested to have improved task performance in an fMRI study of ADHD children with variable intrasubject response patterns during task performance [50]. In the present study, the higher ^99m^Tc-HMPAO uptake in the frontal lobe in the "Depression + ADHD" subgroup compared to in "Depression" may reflect cellular traces of some metabolic process of a more recent onset than the process affecting the cerebellum, or higher brain activity in "Depression + ADHD" to compensate for a "dysmetria of thought" due to a decrease of cerebellar function.

Decreased tracer uptake was found at CBA in "Depression + ADHD" in the caudate nuclei ("cognitive associative striatum") at the comparison with controls, and in the bilateral putamen ("sensorimotor striatum") and thalamus at the comparison with "Depression". A caudate involvement in ADHD has been reported [9]. Decreased activation of the putamen and thalamus has been demonstrated at event-related fMRI in children and adolescents with ADHD [51,52]. An involvement of the putamen and of networks including the thalamus has been suggested in ADHD [9]. A putaminal involvement may reflect various phenomena such as dysfunction of the widespread attention networks due to a disconnection between an anterior cingulate control region with long-range connections and posterior components [34].

The WRAADDS subscale Impulsivity and a Socialization scale were used in the discriminant analysis due to the specific and highly statistical group differences in these parameters. These factors, combined with the neurophysiological measure of the tracer uptake in the thalami, enabled a correct assignment of the cases to the "Depression" and the "Depression + ADHD" subgroups. A high discriminative value of the two scales was expected since the Impulsivity items comprises 10% of the 60 items used at the subgrouping, and since the Socialization items which were formulated 50 years ago deal with aspects of interpersonal difficulties often experienced in ADHD [see Additional file 2]. The fact that limiting the analysis to these scales (with a contribution of the thalami tracer distribution values) allowed for the assignment of all patients to the correct clinical group is of great interest. Kendell and Jablensky, in their discussion on the validity and utility of psychiatric diagnostic criteria and rule-based classifications, suggest that "there may be excellent reasons for using quite different criteria, for example, the presence of a single key symptom; a minimum score on a rating scale; a cognitive, pharmacological, or neurophysiological abnormality, or a combination of these" [53]. It cannot be excluded that the contribution of thalamic tracer uptake in discriminating between the groups reflects more than a spurious finding since decreased thalamic activation has been reported in adolescent ADHD [52].

Evaluating the genders separately may be advantageous in studies using ^99m^Tc-HMPAO SPECT due to observed gender differences using this method [23]. In studies comparing males and females with adult-ADHD, females were more impaired on ADHD scales, had a higher level of emotional symptoms, a more complicated presentation, reported fewer assets and more problems suggesting poorer self-perception, and had increased prevalence of affective disorder and higher somatization scores [5,54,55].

Controlling for age onset and depression severity might not be apt since, in females, more severe symptoms may be inherent characteristics of depression with comorbid adult-ADHD. Inconsistent results concerning age of onset of major depression have been reported in studies of depression with and without adult-ADHD [6,56,57]. No increase of depression severity was observed in a study of depressed patients with comorbid adult-ADHD of whom almost half were male [56]. In females with adolescent and adult-ADHD, major depression was associated with an earlier age at onset, greater than twice the duration, more severe depression-associated impairment, and a higher rate of suicidality than major depression in females without ADHD [57]. The higher depression severity in the "Depression + ADHD" subgroup compared to in "Depression" may thus reflect that only females were studied. However, the disease burden did not differ between "Depression" and "Depression + ADHD", as exemplified by work capacity and other indices of adult life accomplishments (Table 2).

## Limitations

The possibility that medication could have influenced the results cannot be fully ruled out since, although statistically unsignificant, 3/16 (19%) of the "Depression" subgroup patients, and 6/14 (43%) of the "Depression + ADHD" patients, had ongoing anti-depressant medications at the time of SPECT.

It is difficult to evaluate the contribution of the depressive or the audiological symptoms or the heredity for audiological symptoms, in our patients on the brain uptake of ^99m^Tc-HMPAO, and thus the generalisability of our findings to adult-ADHD. No study has to our knowledge been performed reporting the prevalence of uni- or bilateral audiological symptoms of cochlear origin in adult-ADHD. There were no such symptoms in 3/16 (19%) of the patients in the "Depression" subgroup, or in 6/14 (43%) of the "Depression + ADHD" patients, an unsignificant difference. It seems unlikely that the decreased tracer uptake in "Depression + ADHD" compared to controls is related to audiological symptoms since the proportion is lower in "Depression + ADHD". ADHD is more common than expected in deaf children [58]. According to the clinical experience by the first author (A.G.), ADHD in off-spring with normal hearing of depressed patients with uni- or bilateral audiological symptoms of various age onset could be more common than to be expected. Since both audiological and mood disorders are common manifestations of mitochondrial disorders [59], familial co-occurrence is likely to be increased if there is an element of mitochondrial dysfunction in ADHD, as has been suggested [60]. A role of mitochondrial dysfunction in the pathophysiology of bipolar disorder, a condition with familial co-occurrence, symptom overlap and comorbidity with ADHD [61,62], is supported by several findings [43,63,64]. For comments on the issue of a shared pathophysiology between ADHD and comorbid psychiatric and somatic disorders, see [Additional file 1].

In the absence of well-validated and universally accepted diagnostic criteria for adult-ADHD [6,65], our approach using cut-off scores followed by clinical interview may have overdiagnosed as well as underdiagnosed adult-ADHD in the patients in whom no neuropsychological investigation was performed.

## Conclusion

In this study applying the commonly available functional neuro-imaging method SPECT, we found decreased tracer uptake in the bilateral cerebellum in a group of depressed patients with adult-ADHD in comparison with healthy controls as verified by different three-dimensional digitized spatial standardization software. Increased tracer uptake in frontal lobe regions was observed in depressed patients with ADHD in comparison with depressed patients without ADHD tentatively reflecting a difference of the metabolic status in these brain regions which may be caused by some primary biochemical phenomenon, or reflect a compensatory mechanism, between these subgroups of depressed patients. Further studies are warranted exploring if depression with ADHD may represent endophenotype(s) of major depression, i.e., internal phenotypes that are not obvious to the unaided eye filling the gap between available descriptors, and between the gene and the elusive disease process, and therefore may help to resolve questions about etiological models [66].

## List of abbreviations used

^99m^Tc-HMPAO: ^99m^Tc-*d,l*-hexamethylpropylene amine oxime; ADHD: Attention Deficit Hyperactivity Disorder; BA: Brodmann Area; CBA: Computerized Brain Atlas; CBF: Cerebral Blood Flow; DSM: Diagnostic and Statistical Manual of Mental Disorders; fMRI: functional Magnetic Resonance Imaging; KSP: Karolinska Scales of Personality; MADRS-S: Montgomery Aasberg Depression Rating Scale, 9-items version; MPD: Methylphenidate; MRI: Magnetic Resonance Imaging; PET: Positron Emission Tomography; SPECT: Single Photon Emission Computed Tomography; SPM: Statistical Parametric Mapping; VOI: Volumes Of Interest; WURS-25: Wender Utah Retrospective Scale, 25-items version; WRAADDS: Wender-Reimherr Adult Attention Deficit Disorder Scale.

## Competing interests

The authors declare that they have no competing interests.

## Authors' contributions

AG conceived of the study, the recruition and clinical investigation of the subjects, participated in the study design, and drafted the manuscript. DS participated in the methodological and statistical analysis. AV, ASC, HJ and SAL all participated in various parts of the methodological development and evaluation of brain imaging data. SB participated with the design of the neuropsychiatric test battery. MP participated in the design, coordination, statistical analysis and data interpretation of the study, and in the drafting and approval of the manuscript. All authors read and approved the final manuscript.

## Supplementary Material

Additional file 1 **The psychiatric and somatic comorbidity with adult-ADHD**. A table presenting the reported psychiatric and somatic comorbidity with adult-ADHD. The issue of a putative shared pathophysiology is commented. Click here for file

Additional file 2 **The Impulsivity and Socialization scales**. Tables presenting the raw scores of the depressed subgroups on the Impulsivity and Socialization scales that were used in the discrimant analysis, and the individual items. Click here for file
