# Supplementary material for: Differences at brain SPECT between depressed females with and without adult ADHD and healthy controls: etiological considerations
Source: Behav Brain Funct. 2009 Sep 1;5:37. doi: 10.1186/1744-9081-5-37 (PMC2753339; doi:10.1186/1744-9081-5-37)
Supplement: Additional file 1 — The psychiatric and somatic comorbidity with adult-ADHD. A table presenting the reported psychiatric and somatic comorbidity with adult-ADHD. The issue of a putative shared pathophysiology is commented. [file 1744-9081-5-37-S1.doc]

# Supplementary Material for

Differences at brain SPECT between depressed females with and without adult ADHD and healthy controls: etiological considerations

# Additional file 1: The psychiatric and somatic comorbidity with adult-ADHD

**Table S1.** Adult-ADHD (U.S. population prevalence 4.4% [1]) comorbidity with other conditions

| Other childhood onset neuropsychiatric disorders | | Author (first) | year | ref. |
| --- | --- | --- | --- | --- |
| Autism spectrum disorders | adult-ADHD in 38% of 129 autism spectrum disorder patients | Stahlberg | 2004 | [2] |
|  | autism spectrum disorder in 30% of 161 adult-ADHD patients | Stahlberg | 2004 | [2] |
| Character development and personality disorder | |  |  |  |
| Character development | low mean score (< 2 SD) for Self-directedness (i.e., responsible and resourceful vs. blaming and inept) in 100 adult-ADHD patients | Anckarsäter | 2006 | [3] |
| Personality disorder | borderline personality disorder in 37% of 81 adult-ADHD patients | Anckarsäter | 2006 | [3] |
|  | borderline personality disorder in 20% of 152 adult-ADHD patients | Miller | 2007 | [4] |
| Psychiatric disorders | |  |  |  |
| Major depression | adult-ADHD in 12% of 116 patients with major depression | Alpert | 1996 | [5] |
|  | depression in 10% of 51 adult-ADHD patients | Shekim | 1990 | [6] |
|  | depression in 37% of 78 adult-ADHD patients | Downey | 1997 | [7] |
|  | depression in 17% of 2252 adult-ADHD subjects | Secnik | 2005 | [8] |
|  | depression in 19% of 154 adult-ADHD subjects | Kessler | 2006 | [1] |
|  | depression risk 2.5 times higher in adult-ADHD, sample 262 females | Biederman | 2008 | [9] |
| Bipolar disorder | adult-ADHD in 16% of 159 bipolar disorder-I patients | Tamam | 2007 | [10] |
|  | adult-ADHD in 21% of bipolar disorder-subjects | Kessler | 2006 | [1] |
|  | bipolar disorder in 47% of 51 adult-ADHD patients | Wilens | 2003 | [11] |
|  | bipolar disorder in 5% of 79 adult-ADHD patients | McGough | 2005 | [12] |
|  | bipolar disorder in 4% of 2252 adult-ADHD subjects | Secnik | 2005 | [8] |
|  | bipolar disorder in 19% of 154 adult-ADHD subjects | Kessler | 2006 | [1] |
|  | bipolar disorder in 18% of 127 adult-ADHD patients | Faraone | 2006 | [13] |
| Any mood disorder | lifetime mood disorder in 53% of 152 patients with adult-ADHD | Miller | 2007 | [4] |
| Anxiety disorder | anxiety disorder in 51% of 51 adult-ADHD patients | Shekim | 1990 | [6] |
|  | anxiety disorders in 14% of 2252 adult-ADHD subjects | Secnik | 2005 | [8] |
|  | anxiety disorder in 47% of 154 adult-ADHD subjects | Kessler | 2006 | [1] |
|  | lifetime anxiety disorder in 30% of 152 adult-ADHD patients | Miller | 2007 | [4] |
| Somatic disorders | |  |  |  |
| Restless legs syndrome | adult-ADHD in 24% of 62 RLS patients* | Wagner | 2004 | [14] |
| Obesity | adult-ADHD in 27% of 215 females with obesity | Altfas | 2002 | [15] |
|  | adult-ADHD in 27% of 75 severely obese females | Fleming | 2005 | [16] |
| Fibromyalgia | adult-ADHD prevalence suggested to be increased in fibromyalgia | Gillberg | 2005 | [17] |
|  | fibromyalgia prevalence suggested to be increased in adult-ADHD | Young | 2007 | [18] |

* Up to 44% of children with ADHD have been found to have restless legs syndrome (RLS) or RLS symptoms [19]. The cause of RLS is unknown but the finding that RLS is associated with several factors including the presence of a mental disorder and obesity [20], migraine [21-23], and fibromyalgia [24-26] suggest *a shared pathophysiology*. Capillary dilatations and irregular capillary formations in nailfold have been reported in fibromyalgia [27]. Other findings in fibromyalgia indicate abnormal mitochondrial energy production [28]. Aerobic performance and muscle biopsies of 20 RLS patients compared to 16 controls revealed lower oxygen uptake and increased capillary tortuosity in the RLS patients [29]. The capillary alterations indicate histotoxic ischemia which can be caused by oxygen deficiency or “chemical/metabolic ischemia”. The latter may be due to genetic mechanisms or effects of cofactor deficiency or toxins on the metabolism of the cellular-energy producing mitochondrial respiratory chain consuming the bulk of inhaled oxygen.

*A shared pathophysiology* may be present for ADHD and its comorbid conditions. Elevated plasma lactate levels, a sign of mitochondrial disease, were found in 14 of 69 patients (20%) with autism, and definite mitochondrial respiratory chain disorder in five (7%) suggesting that mitochondrial dysfunction is one of the most common medical conditions associated with autism [30]. The findings have been supported by further studies by the same team [31,32], and by another team [33]. Studies of bipolar disorder have revealed elevated cerebrospinal fluid lactate consistent with impaired mitochondrial metabolism [34] and altered expression levels of subunits of a mitochondrial enzyme complex in the brain [35]. Although bipolar disorder did not seem to be a classic mitochondrial disease, subtle deficits in mitochondrial function have been proposed to play an important role in the neuroplasticity of bipolar disorder [36]. Carnitine, an agent necessary for mitochondrial function, has been reported to ameliorate symptoms of ADHD/ADD in two multicenter double-blind placebo controlled studies [37,38] supporting the proposal that deficient cellular energy production may underlie some of the observed neurophysiological and psychophysiological abnormalities in ADHD [39].

## References

1. Kessler RC, Adler L, Barkley R, Biederman J, Conners CK, Demler O, Faraone SV, Greenhill LL, Howes MJ, Secnik K, Spencer T, Ustun TB, Walters EE, Zaslavsky AM: **The prevalence and correlates of adult ADHD in the United States: results from the National Comorbidity Survey Replication.** *Am J Psychiatry* 2006, **163:**716-723.
2. Stahlberg O, Söderström H, Råstam M, Gillberg C: **Bipolar disorder, schizophrenia, and other psychotic disorders in adults with childhood onset AD/HD and/or autism spectrum disorders.** *J Neural Transm* 2004, **111:**891-902.
3. Anckarsäter H, Stahlberg O, Larson T, Håkansson C, Jutblad SB, Niklasson L, Nydén A, Wentz E, Westergren S, Cloninger CR, Gillberg C, Råstam M: **The impact of ADHD and autism spectrum disorders on temperament, character, and personality development.** *Am J Psychiatry* 2006, **163:**1239-1244.
4. Miller TW, Nigg JT, Faraone SV: **Axis I and II comorbidity in adults with ADHD.** *J Abnorm Psychol* 2007, **116:**519-528.
5. Alpert JE, Maddocks A, Nierenberg AA, O'Sullivan R, Pava JA, Worthington JJ 3rd, Biederman J, Rosenbaum JF, Fava M: **Attention deficit hyperactivity disorder in childhood among adults with major depression.** *Psychiatry Res* 1996, **62:**213-219.
6. Shekim WO, Asarnow RF, Hess E, Zaucha K, Wheeler N: **A clinical and demographic profile of a sample of adults with attention deficit hyperactivity disorder, residual state.** *Compr Psychiatry* 1990, **31:**416-425.
7. Downey KK, Stelson FW, Pomerleau OF, Giordani B: **Adult attention deficit hyperactivity disorder: psychological test profiles in a clinical population.** *J Nerv Ment Dis* 1997, **185:**32-38.
8. Secnik K, Swensen A, Lage MJ: **Comorbidities and costs of adult patients diagnosed with attention-deficit hyperactivity disorder.** *Pharmacoeconomics* 2005, **23:**93-102.
9. Biederman J, Ball SW, Monuteaux MC, Mick E, Spencer TJ, McCreary M, Cote M, Faraone SV: **New insights into the comorbidity between ADHD and major depression in adolescent and young adult females.** *J Am Acad Child Adolesc Psychiatry* 2008, **47:**426-434.
10. Tamam L, Karakus G, Ozpoyraz N: **Comorbidity of adult attention-deficit hyperactivity disorder and bipolar disorder: prevalence and clinical correlates.** *Eur Arch Psychiatry Clin Neurosci* 2008, **258:**385-393.
11. Wilens TE, Biederman J, Spencer TJ: **Attention deficit/hyperactivity disorder across the lifespan.** *Annu Rev Med* 2002, **53:**113-131.
12. McGough JJ, Smalley SL, McCracken JT, Yang M, Del'Homme M, Lynn DE, Loo S: **Psychiatric comorbidity in adult attention deficit hyperactivity disorder: findings from multiplex families.** *Am J Psychiatry* 2005, **162:**1621-1627.
13. Faraone SV, Biederman J, Spencer T, Mick E, Murray K, Petty C, Adamson JJ, Monuteaux MC: **Diagnosing adult attention deficit hyperactivity disorder: are late onset and subthreshold diagnoses valid?** *Am J Psychiatry* 2006, **163:**1720-1729; quiz 1859.
14. Wagner ML, Walters AS, Fisher BC: **Symptoms of attention-deficit/hyperactivity disorder in adults with restless legs syndrome.** *Sleep* 2004, ;27:1499-1504.
15. Altfas JR: **Prevalence of attention deficit/hyperactivity disorder among adults in obesity treatment.** *BMC Psychiatry* 2002, **2:**9.
16. Fleming JP, Levy LD, Levitan RD: **Symptoms of attention deficit hyperactivity disorder in severely obese women.** *Eat Weight Disord* 2005, 10:e10-13.
17. Gillberg C, Gillberg IC, Rasmussen P, Kadesjö B, Söderström H, Råstam M, Johnson M, Rothenberger A, Niklasson L: **Co-existing disorders in ADHD -- implications for diagnosis and intervention.** *Eur Child Adolesc Psychiatry* 2004, **13 Suppl 1:**I80-I92.
18. Young JL, Redmond JC: **Fibromylagia, chronic fatigue, and adult attention deficit hyperactivity disorder in the adult: a case study.** *Psychopharmacol Bull* 2007, **40:**118-126.
19. Cortese S, Konofal E, Lecendreux M, Arnulf I, Mouren MC, Darra F, Dalla Bernardina B: **Restless legs syndrome and attention-deficit/hyperactivity disorder: a review of the literature.** *Sleep* 2005, **28:**1007-1013.
20. Ohayon MM, Roth T: **Prevalence of restless legs syndrome and periodic limb movement disorder in the general population.** *J Psychosom Res* 2002, **53:**547-554.
21. Rhode AM, Hösing VG, Happe S, Biehl K, Young P, Evers S: **Comorbidity of migraine and restless legs syndrome--a case-control study.** Cephalalgia 2007, **27:**1255-1260.
22. Sabayan B, Bagheri M, Borhani Haghighi A: **Possible joint origin of restless leg syndrome (RLS) and migraine.** *Med Hypotheses* 2007, **69:**64-66.
23. d'Onofrio F, Bussone G, Cologno D, Petretta V, Buzzi MG, Tedeschi G, Bonavita V, Cicarelli G: **Restless legs syndrome and primary headaches: a clinical study.** *Neurol Sci* 2008, **29 Suppl 1:**S169-S172.
24. Yunus MB, Aldag JC: **Restless legs syndrome and leg cramps in fibromyalgia syndrome: a controlled study.** *BMJ* 1996, **312:**1339.
25. Tayag-Kier CE, Keenan GF, Scalzi LV, Schultz B, Elliott J, Zhao RH, Arens R: **Sleep and periodic limb movement in sleep in juvenile fibromyalgia.** *Pediatrics* 2000, 106:E70.
26. Shaver JL, Wilbur J, Robinson FP, Wang E, Buntin MS: **Women's health issues with fibromyalgia syndrome.** *J Womens Health (Larchmt)* 2006, **15:**1035-1045.
27. Morf S, Amann-Vesti B, Forster A, Franzeck UK, Koppensteiner R, Uebelhart D, Sprott H: **Microcirculation abnormalities in patients with fibromyalgia - measured by capillary microscopy and laser fluxmetry.** *Arthritis Res Ther* 2005, **7:**R209-R216.
28. Sprott H, Salemi S, Gay RE, Bradley LA, Alarcón GS, Oh SJ, Michel BA, Gay S: **Increased DNA fragmentation and ultrastructural changes in fibromyalgic muscle fibres.** *Ann Rheum Dis* 2004, **63:**245-251.
29. Wahlin Larsson B, Kadi F, Ulfberg J, Piehl Aulin K: **Skeletal muscle morphology in patients with Restless Legs Syndrome.** *Eur Neurol* 2007, **58:**133-137.
30. Oliveira G, Diogo L, Grazina M, Garcia P, Ataíde A, Marques C, Miguel T, Borges L, Vicente AM, Oliveira CR: **Mitochondrial dysfunction in autism spectrum disorders: a population-based study.** *Dev Med Child Neurol* 2005, **47:**185-189.
31. Correia C, Coutinho AM, Diogo L, Grazina M, Marques C, Miguel T, Ataíde A, Almeida J, Borges L, Oliveira C, Oliveira G, Vicente AM: Brief report: **High frequency of biochemical markers for mitochondrial dysfunction in autism: no association with the mitochondrial aspartate/glutamate carrier SLC25A12 gene.** *J Autism Dev Disord* 2006, **36:**1137-1140.
32. Oliveira G, Ataíde A, Marques C, Miguel TS, Coutinho AM, Mota-Vieira L, Gonçalves E, Lopes NM, Rodrigues V, Carmona da Mota H, Vicente AM: **Epidemiology of autism spectrum disorder in Portugal: prevalence, clinical characterization, and medical conditions.** *Dev Med Child Neurol* 2007, **49:**726-733.
33. Weissman JR, Kelley RI, Bauman ML, Cohen BH, Murray KF, Mitchell RL, Kern RL, Natowicz MR: **Mitochondrial disease in autism spectrum disorder patients: a cohort analysis.** *PLoS ONE* 2008, **3:**e3815.
34. Regenold WT, Phatak P, Marano CM, Sassan A, Conley RR, Kling MA: **Elevated cerebrospinal fluid lactate concentrations in patients with bipolar disorder and schizophrenia: Implications for the mitochondrial dysfunction hypothesis.** *Biol Psychiatry* 2009, **65:**489-494.
35. Ben-Shachar D, Karry R: **Neuroanatomical pattern of mitochondrial complex I pathology varies between schizophrenia, bipolar disorder and major depression.** *PLoS ONE* 2008;**3:**e3676.
36. Quiroz JA, Gray NA, Kato T, Manji HK: **Mitochondrially mediated plasticity in the pathophysiology and treatment of bipolar disorder.** *Neuropsychopharmacology* 2008, **33:**2551-2565.
37. Arnold LE, Amato A, Bozzolo H, Hollway J, Cook A, Ramadan Y, Crowl L, Zhang D, Thompson S, Testa G, Kliewer V, Wigal T, McBurnett K, Manos M: **Acetyl-L-carnitine (ALC) in attention-deficit/hyperactivity disorder: a multi-site, placebo-controlled pilot trial.** *J Child Adolesc Psychopharmacol* 2007, **17:**791-802.
38. Torrioli MG, Vernacotola S, Peruzzi L, Tabolacci E, Mila M, Militerni R, Musumeci S, Ramos FJ, Frontera M, Sorge G, Marzullo E, Romeo G, Vallee L, Veneselli E, Cocchi E, Garbarino E, Moscato U, Chiurazzi P, D'Iddio S, Calvani M, Neri G: **A double-blind, parallel, multicenter comparison of L-acetylcarnitine with placebo on the attention deficit hyperactivity disorder in fragile X syndrome boys.** *Am J Med Genet A* 2008, **146:**803-812.
39. Russell VA, Oades RD, Tannock R, Killeen PR, Auerbach JG, Johansen EB, Sagvolden T: **Response variability in Attention-Deficit/Hyperactivity Disorder: a neuronal and glial energetics hypothesis.** *Behav Brain Funct* 2006, **23:**2-30.
