# Supplementary material for: Differences at brain SPECT between depressed females with and without adult ADHD and healthy controls: etiological considerations
Source: Behav Brain Funct. 2009 Sep 1;5:37. doi: 10.1186/1744-9081-5-37 (PMC2753339; doi:10.1186/1744-9081-5-37)
Supplement: Additional file 2 — The Impulsivity and Socialization scales. Tables presenting the raw scores of the depressed subgroups on the Impulsivity and Socialization scales that were used in the discrimant analysis, and the individual items. [file 1744-9081-5-37-S2.doc]

# *Supplementary Material for*

Differences at brain SPECT between depressed females with and without adult ADHD and healthy controls: etiological considerations

# Additional file 2: The Impulsivity and Socialization scales

The WRAADDS subscale Impulsivity and the KSP subscale Socialization were used in the discriminant analysis due to the specific and highly statistical group differences in these parameters. These factors, combined with the neurophysiological measure of the tracer uptake in the thalami, enabled a correct assignment of the cases to the “Depression” and “Depression + ADHD” groups. In our study, the subscales were answered in the context of the full questionnaires. The individual items of the Impulsivity and Socialization subscales are presented in this additional file since they may be of use as assisting screening instruments at differential diagnosis of adult-ADHD in depression, see Tables S3 and S6 on page 2 and 4.

**The 6 Impulsivity items**

The scores of the 6 items differed significantly (p < 0.0001) between the ”Depression” and ”Depression + ADHD” depressed patients, see Table S2. The items are presented in Table S3.

**Table S2.** Mean Impulsivity scores in the depressed subgroups

| “Depression” | 3 ± 2 |
| --- | --- |
| ”Depression + ADHD” | 15 ± 4 |

**Table S3.**

| Put a cross in the square that fits the best for how you usually react or think your situation is.  There are five possible answers:  *0 = Not present*  *1 = Mild*  *2 = Moderate*  *3 = Quite a bit*  *4 = Very much* | Not present | Mild | Moderate | Quite a bit | Very much |
| --- | --- | --- | --- | --- | --- |
| I am a dare-devil | 0 | 1 | 2 | 3 | 4 |
| I often act on the spur of the moment without stopping to think about the consequences, and particularly if I am angry | 0 | 1 | 2 | 3 | 4 |
| I tend to interrupt people when they are talking | 0 | 1 | 2 | 3 | 4 |
| I get into trouble because I talk without first thinking | 0 | 1 | 2 | 3 | 4 |
| I am impatient | 0 | 1 | 2 | 3 | 4 |
| I tend to act first and to think (and later) regret | 0 | 1 | 2 | 3 | 4 |

**The 20 Socialization items**

The scores of the 20 items differed significantly (p < 0.0001) between the ”Depression” and the ”Depression + ADHD” depressed patients, see Table S4. Low scores indicate pathology. The conversion of raw scores to T-scores is presented in Table S8.

**Table S4.** Mean T-scores and raw scores in the depressed subgroups

|  | T-score* | Raw scores | | |
| --- | --- | --- | --- | --- |
|  | All ages | Age 20 - 34 | Age 35 - 49 | Age 50 - 65 |
| “Depression” | 43 ± 10 | 60 ± 9 | 61 ± 8 | 63 ± 8 |
| ”Depression + ADHD” | 22 ± 10 | 42 ± 9 | 43 ± 8 | 46 ± 8 |

*50 ± 10 reflects the normal mean.

The KSP Socialization items were selected from the Gough Socialization scale [1] and are presented in Table S6. Conceptual analysis classified the selected items into three subgroups which was later confirmed by factor analysis: 10 items have a similar face validity of negative childhood experiences, three items are related to school maladjustment, and the common denominator for the remaining seven items is conceptualized as a present feeling of resentment and victimization [2]. Numbering according to the 135-items KSP in the left hand column in light grey in order to be less obvious at printing. Reversed scoring, see Table S5, is signified by a minus sign before the KSP item number and is used apart from in items 32, 50, 58 and 125 (the 5^th^, 8^th^, 9^th^ and 19^th^ items).

**Table S5.** Normal and reversed scoring

| Normal scoring | Reversed scoring | Score |
| --- | --- | --- |
| Does not apply at all | Applies completely | 1 |
| Does not apply very well | Applies rather well | 2 |
| Applies rather well | Does not apply very well | 3 |
| Applies completely | Does not apply at all | 4 |

Table S6.

| KSP item | Put a cross in the square that fits the best for how you usually react or think your situation is, or how it used to be.  There are four possible answers:  *Does not apply at all*  *Does not apply very well*  *Applies rather well*  *Applies completely* | Does not apply at all | *Does not apply very well* | Applies rather well | Applies completely |
| --- | --- | --- | --- | --- | --- |
| - 5 | I have had more than my share of things to worry about |  |  |  |  |
| - 10 | As a child, I sometimes used to feel that I would like to leave home |  |  |  |  |
| - 18 | My parents have often disapproved of my friends |  |  |  |  |
| - 24 | Life usually hands me a pretty raw deal |  |  |  |  |
| 32 | My home life was always happy |  |  |  |  |
| - 37 | I have often gone against my parents wishes |  |  |  |  |
| - 45 | I have a feeling that people oftentimes talk about me behind my back |  |  |  |  |
| 50 | My home life was always very pleasant |  |  |  |  |
| 58 | The members of my family were always very close to each other |  |  |  |  |
| - 64 | My home as a child was less peaceful and quiet than those of most other people |  |  |  |  |
| - 72 | In school I was sometimes sent to the principal for cutting up |  |  |  |  |
| - 77 | As a child, I sometimes wanted to run away from home |  |  |  |  |
| - 85 | I was not responsible for most of my troubles |  |  |  |  |
| - 90 | With things going as they are, it's pretty hard to keep up hope of amounting to something |  |  |  |  |
| - 98 | As a youngster in school I used to give the teachers lots of trouble |  |  |  |  |
| -104 | My parents never really understood me |  |  |  |  |
| -112 | I seem to do things that I regret more often than other people do |  |  |  |  |
| -117 | When I was going to school I played hooky quite often |  |  |  |  |
| 125 | My parents generally let me make my own decisions |  |  |  |  |
| -131 | I often feel as though I have done something wrong or wicked |  |  |  |  |

**Table S7.** The female Stockholm population raw means (M) and Standard Deviations (SD) [3]

| Age years | M | SD |
| --- | --- | --- |
| 20 - 34 | 66.1 | 8.6 |
| 35 - 49 | 66.6 | 8.3 |
| 50 - 65 | 68.6 | 8.1 |

X – M

Conversion of patient´s KSP raw scores (X) into T-scores: ---------------- x 10 + 50

SD

**Table S8. Conversion table of raw scores to T-scores for the Socialization scale**

| Females | T-scores | | |
| --- | --- | --- | --- |
| Raw scores | Age 20 - 34 | Age 35 - 49 | Age 50 - 65 |
| 23 |  |  |  |
| 24 | 1 |  |  |
| 25 | 2 | 0 |  |
| 26 | 3 | 1 |  |
| 27 | 5 | 2 |  |
| 28 | 6 | 3 | 0 |
| 29 | 7 | 5 | 1 |
| 30 | 8 | 6 | 2 |
| 31 | 9 | 7 | 4 |
| 32 | 10 | 8 | 5 |
| 33 | 12 | 10 | 6 |
| 34 | 13 | 11 | 7 |
| 35 | 14 | 12 | 9 |
| 36 | 15 | 13 | 10 |
| 37 | 16 | 14 | 11 |
| 38 | 17 | 16 | 12 |
| 39 | 18 | 17 | 13 |
| 40 | 20 | 18 | 15 |
| 41 | 21 | 19 | 16 |
| 42 | 22 | 20 | 17 |
| 43 | 23 | 22 | 18 |
| 44 | 24 | 23 | 20 |
| 45 | 25 | 24 | 21 |
| 46 | 27 | 25 | 22 |
| 47 | 28 | 26 | 23 |
| 48 | 29 | 28 | 25 |
| 49 | 30 | 29 | 26 |
| 50 | 31 | 30 | 27 |
| 51 | 32 | 31 | 28 |
| 52 | 34 | 32 | 30 |
| 53 | 35 | 34 | 31 |
| 54 | 36 | 35 | 32 |
| 55 | 37 | 36 | 33 |
| 56 | 38 | 37 | 34 |
| 57 | 39 | 38 | 36 |
| 58 | 41 | 40 | 37 |
| 59 | 42 | 41 | 38 |
| 60 | 43 | 42 | 39 |
| 61 | 44 | 43 | 41 |
| 62 | 45 | 44 | 42 |
| 63 | 46 | 46 | 43 |
| 64 | 48 | 47 | 44 |
| 65 | 49 | 48 | 46 |
| 66 | 50 | 49 | 47 |
| 67 | 51 | 50 | 48 |
| 68 | 52 | 52 | 49 |
| 69 | 53 | 53 | 50 |
| 70 | 55 | 54 | 52 |
| 71 | 56 | 55 | 53 |
| 72 | 57 | 57 | 54 |
| 73 | 58 | 58 | 55 |
| 74 | 59 | 59 | 57 |
| 75 | 60 | 60 | 58 |
| 76 | 62 | 61 | 59 |
| 77 | 63 | 63 | 60 |
| 78 | 64 | 64 | 62 |
| 79 | 65 | 65 | 63 |
| 80 | 66 | 66 | 64 |

##### References

1. Gough HG: **Theory and measurement of socialization.** *J Consult Psychol* 1960, **24:**23-30.
2. Svanborg P, Gustavsson PJ, Mattila-Evenden M, Åsberg M: **Assessment of maladaptiveness: a core issue in the diagnosing of personality disorders.** *J Personal Disord* 1999, **13:**241-256.
3. Bergman H. Bergman I. Engelbrekson K. Holm L. Johannesson K. Lindberg S: *Psykologhandbok.* Del I (Psychological Manual. Part I). Magnus Huss Klinik. Stockholm; 1982.
